# Inhibition of phosphatase and tensin homologue (PTEN) in human ovary *in vitro* results in increased activation of primordial follicles but compromises development of growing follicles

**DOI:** 10.1093/molehr/gau037

**Published:** 2014-05-15

**Authors:** Marie McLaughlin, Hazel L. Kinnell, Richard A. Anderson, Evelyn E. Telfer

**Affiliations:** 1 Institute of Cell Biology and Centre for Integrative Physiology, University of Edinburgh, Hugh Robson Building, George Square, Edinburgh EH8 9XD, UK; 2 Medical Research Council Centre for Reproductive Health, Queen's Medical Research Institute, University of Edinburgh, 47 Little France Crescent, Edinburgh EH16 4TJ, UK

**Keywords:** PTEN, *in vitro*, human, follicle, ovary

## Abstract

In the mammalian ovary a small number of follicles are steadily recruited from the quiescent pool to undergo development. Follicle loss, maintenance and growth are strictly controlled by complex molecular interactions including the phosphoinositide 3-kinase (PI3K)-protein kinase B (Akt) signalling pathway. Stimulation of PI3K promotes phosphorylation of Akt resulting in follicle survival and activation of growth whereas this pathway is suppressed by the actions of the phosphatase and tensin homologue (PTEN). The aim of this study was to determine the effect of dipotassium bisperoxo(5-hydroxypyridine-2-carboxyl)oxovanadate (bpV), a reversible inhibitor of PTEN, on the activation, survival and development of human ovarian follicles *in vitro*. Biopsied ovarian tissue fragments were obtained from 17 women aged 23–46 years and exposed to 1 µM bpV(HOpic) (*n* = 146) or control medium (*n* = 128) for 24 h. Media were then replaced with control medium and all tissue incubated for a further 5 days. Ovarian tissue from each treatment group was fixed after the initial 24 h culture period and phosphorylated Akt was quantified by western blotting. After 6 days incubation all tissue fragments were inspected under light microscopy and any secondary follicles ≥100 µm isolated. Isolated follicles were cultured individually in control medium supplemented with 100 ng/ml recombinant human activin A. Tissue fragments without follicles suitable for isolation were fixed and processed for histological and immunohistochemical analysis. During 6 days culture, follicle activation occurred in tissue samples from both treatment groups but with significantly more follicles progressing to the secondary stage of development in the presence of 1 µM bpV(HOpic) compared with control (31 versus 16%; *P* < 0.05). Increased activation was associated with increased Akt phosphorylation and increased nuclear export of FOXO3. However isolated and cultured follicles that had been exposed to bpV(HOpic) showed limited growth and reduced survival compared with follicles from control fragments (*P* < 0.05). This study demonstrates that inhibition of PTEN with bpV(HOpic) affects human ovarian follicle development by promoting the initiation of follicle growth and development to the secondary stage, as in rodent species, but severely compromises the survival of isolated secondary follicles.

## Introduction

Human ovarian follicles largely exist as a quiescent population, of which a small number daily initiate growth throughout reproductive life. Only a small proportion of these follicles go on to complete growth and release a mature fertilizable oocyte. Remaining follicles degenerate either from the dormant state (Tingen *et al.*, 2009) or after growth has been initiated (Kaipia and Hsueh, 1997). Development of growing follicles is controlled by the coordinated actions of multiple complex, integrated signalling pathways regulated by local and systemic hormonal signals (Pangas, 2007; Sobinoff *et al.*, 2013) and reproductive senescence occurs when the quiescent follicle population is exhausted through activation and degeneration. Prior to exhaustion of the follicle pool, the overwhelming majority of human follicles are dormant and can persist in this state for decades. The ability to recruit these dormant follicles into the growing pool and support their complete development *in vitro* would address the scarcity of oocytes available for assisted reproduction techniques (ART), fertility preservation and provide basic scientific information on human germ cell development.

Biochemical and genetic manipulation studies in the mouse have identified the phosphoinositide 3-kinase - protein kinase B (PI3K-Akt) signalling pathway as a key mechanism involved in the maintenance of follicle growth and loss. Several growth factors, including follicle stimulating hormone (FSH), stimulate PI3K to activate phosphoinositide-dependent protein kinase-1 (PDK1) resulting in phosphorylation of Akt and downstream transcription factors including the forkhead winged helix box O1 (FOXO1) triggering follicle activation and development (Gonzalez-Robayna *et al.*, 2000; Alum *et al.*, 2004). These events are reversed by the action of phosphatase and tensin homologue (PTEN) which causes dephosphorylation of phosphatidlyinositol 3, 4, 5-triphosphate (PIP3) thus regulating the initiation of follicle growth and preventing premature exhaustion of the follicle pool (Cantley and Neel, 1999; John *et al.*, 2008). The effect of PTEN can be reversibly inhibited by vanadate derivatives acting as protein tyrosine phosphatase inhibitors thereby promoting the downstream phosphorylation of Akt (Schmid *et al.*, 2004; Morohaku *et al.*, 2013). Recent studies have demonstrated the importance of the PTEN and the PI3K pathway within the oocyte in the regulation of murine ovarian follicle activation. Deletion of *PTEN* in mouse oocytes resulted in pan-ovarian follicle activation and premature oocyte depletion whereas disruption of granulosa cell-specific *PTEN* did not affect initiation of follicle growth but increased granulosa cell proliferation and enhanced ovulation (Fan *et al.*, 2008; Reddy *et al.*, 2008). Li et al*.* (2010) investigated the effect of exposing whole mouse ovaries to a combination of a vanadate derivative compound dipotassium bisperoxo(5-hydroxypyridine-2-carboxyl)oxovanadate (V) (bpV(HOpic)), and 740Y-P, a cell-permeable phospho-peptide PI3K pathway promoter. These experiments demonstrated increased activation of dormant follicles evidenced by increased ovarian weight, the histological detection of large antral follicles and immunohistological detection of nuclear exclusion of forkhead box O3 protein (FOXO3) in oocytes. After bpV(HOpic) treatment, ovaries grafted under the kidney capsule were able to complete development and generate mature eggs (Li *et al.*, 2010). Moreover using a xeno-transplantation model human ovarian follicles treated with bpV(HOpic) developed into pre-ovulatory follicles with oocytes that appeared to be capable of undergoing *in vitro* maturation (Li *et al.*, 2010). It has been suggested that PTEN inhibitors could be used to generate mature oocytes in women whose oocyte reserve is impaired due to illness or treatment and to provide oocytes for derivation of embryonic stem cells (Li *et al.*, 2010; Adhikari *et al.*, 2012). Investigation of the human follicle response to bpV(HOpic) has so far required a xeno-transplantation model to complete follicle development. Therefore, it is not yet known how promotion of the PI3K pathway affects the development of *in vitro* grown (IVG) human ovarian follicles or whether chemically enhanced initiation of follicle growth could provide a population of biopsy-derived growing follicles of sufficient quality for *in vitro* maturation.

Culture systems have been designed to support the key stages of human follicle development. The initiation of follicle growth (Hovatta *et al.*, 1997; Abir *et al.*, 1999; Hovatta *et al.*, 1999; Wright *et al.*, 1999; Picton and Gosden, 2000; Zhang *et al.*, 2004; Telfer *et al.*, 2008), primary to secondary transition (Abir *et al.*, 1997, 1999; Telfer *et al.*, 2008), pre-antral follicle development (Roy and Treacy, 1993; Abir *et al.*, 1997; Otala *et al.*, 2004; Telfer *et al.*, 2008; Xu *et al.*, 2009) and oocyte maturation (Shea *et al.*, 1975; Alak *et al.*, 1998; Mikkelsen *et al.*, 1998) have all been achieved *in vitro*. Despite the prevalence of primordial follicles in the ovary (Gougeon 1986; Gosden and Telfer, 1987) development of systems supporting the continuum of initiation of growth through to final oocyte maturation *in vitro* has been hampered by the limited availability of human ovarian tissue and the recognized variation in follicle density between and within cortical biopsies (Kohl *et al.*, 2000; Qu *et al.*, 2000; Poirot *et al.*, 2002; Schmidt *et al.*, 2003). The purpose of this study was to investigate the effect of inhibition of PTEN on the initiation of human follicle growth and the subsequent survival and development of follicles *in vitro,* by incubating ovarian cortical fragments in the presence of the phosphatase inhibitor bpV(HOpic). This approach uses a two-step culture system designed to promote the activation of human follicle growth within fragments of ovarian cortex and then support the continued development of isolated secondary follicles.

## Materials and Methods

### Ovarian cortical tissue

Cortical tissue was obtained by ovarian biopsy from a total of 17 adult women; 13 undergoing elective Caesarean section and 4 who underwent laparoscopy for non-malignant gynaecological conditions. Their mean (±SD) age was 36.5 ± 1.3 years, ranging from 23 to 46 years. This study received local ethical committee approval and all women gave informed consent.

### Tissue preparation and fragment culture

Ovarian cortex was transferred to the laboratory in pre-warmed dissection medium [Leibovitz medium (Life Technologies Ltd, Paisley, UK) supplemented with sodium pyruvate (2 mM), glutamine (2 mM) (both Life Technologies Ltd), human serum albumin (HSA) (3 mg/ml), penicillin G (75 µg/ml) and streptomycin (50 µg/ml) (Sigma-Aldrich Chemicals, Dorset, UK)]. The biopsied tissue pieces were transferred into fresh dissection medium under laminar flow conditions and examined carefully using light microscopy to distinguish cortical tissue from the underlying stroma. Damaged and/or haemorrhagic tissue was excised allowing the pieces to flatten. With the cortex uppermost the tissue was gently stretched using the blunt edge of a scalpel blade and excess stromal tissue removed. Then using an angled incision the tissue was cut into fragments of ∼4 × 2 × 1 mm thick. Using a dissecting microscope, each fragment was examined for the presence of follicles. A mean diameter was recorded for each follicle observed. Any follicles measuring >40 µm were excised from the tissue fragments using either 25 gauge needles attached to 1 ml syringe barrels or a no.10 blade and handle and fine forceps; this was to ensure a presumptive population of unilaminar follicles. Three to four fragments were selected from each biopsy as 0 h controls and fixed in 10% buffered formalin (NBF) for histological evaluation. The remaining pieces were cultured individually in culture medium [McCoy's 5a medium with bicarbonate supplemented with HEPES (25 mM) (Life Technologies Ltd), glutamine (3 mM) (Life Technologies Ltd), HSA (0.1%), penicillin G (0.1 mg/ml), streptomycin (0.1 mg/ml), transferrin (2.5 µg/ml), selenium (4 ng/ml) and ascorbic acid (50 µg/ml) (all obtained from Sigma-Aldrich Chemicals, Dorset, UK, unless otherwise stated)] or culture medium supplemented with 1 µM bpV(HOpic) (Merck Chemicals Ltd, UK) at 37°C in humidified air with 5% CO_2_ for 24 h. Half the tissue fragments from both groups were then snap-frozen and stored at −80°C for western blot analysis. Medium was removed from the remaining fragments and replaced with fresh culture medium without bpV(HOpic) and supplemented with insulin (10 ng/ml) (Sigma-Aldrich Chemicals) and hFSH (1 ng/ml) (Sigma-Aldrich Chemicals). Tissue was incubated for a further 5 days with half the medium being removed and replaced every second day. A total of 274 cortical fragments were cultured from 17 separate ovarian biopsies (control *n* = 128; 1 µM bpV(HOpic) *n* = 146).

### Follicle isolation and culture

After 6 days of incubation tissue fragments were transferred to dissection medium and examined under light microscopy. Follicles were inspected using a dissecting microscope and a mean diameter for each was calculated. Secondary follicles ≥100 µm in diameter were dissected using 25 gauge needles. Tissue fragments with no detectable follicles or only unilaminar follicles present were fixed in NBF and processed as described below. A total of 51 pre-antral follicles were isolated from tissue fragments incubated in control medium or medium supplemented with 1 µM bpV(HOpic). Isolated follicles were placed individually in 96-well V-bottomed culture plates in 150 µl of culture medium supplemented with insulin (10 ng/ml), hFSH (1 ng/ml) and 100 ng/ml recombinant human activin A (rhAct A) (R & D Systems, Abingdon, UK). Isolated follicles were incubated for a further 6 days at 37°C in humidified air with 5% CO_2_. Every second day half the culture medium was removed and replaced and at the same time follicle diameter was measured using a dissecting microscope with a crossed micrometer.

### Tissue processing

On completion of the incubation period isolated follicles and fragments of cultured cortical tissue were fixed for 24 h in 10% neutral buffered formalin NBF and dehydrated in increasing concentrations of ethanol (70, 90 and 100%). Absolute alcohol was removed and replaced with cedar wood oil (BDH Laboratory Supplies, Poole, UK) for 24 h. The tissue was removed to toluene (Fisher Scientific UK Ltd, Loughborough, UK) for 30 min to ensure complete clearance of oil. Isolated follicles/cortical fragments were individually embedded in paraffin wax at 60°C for 4 h with hourly changes of wax to ensure complete removal of toluene. Isolated follicles and cortical fragments were cut into 6 µm sections, mounted on gelatin-coated slides and left to dry overnight prior to staining with haematoxylin and eosin. Uncultured tissue fragments collected from each biopsy were processed also for histological analysis using the same methodology.

### Immunohistochemistry

bpV(HOpic) and control treated tissue fragments were fixed in NBF, dehydrated in alcohol, embedded in paraffin wax and cut into 6 µm sections as described above to investigate the expression of FOXO3. Antigen retrieval was performed using 0.01 M sodium citrate for 20 min and endogenous peroxidase activity was quenched using 3% hydrogen peroxide in methanol. Tissue sections were incubated in FOXO3 #9467s monoclonal primary antibody (Cell Signalling, Herts, UK) overnight at 4°C. Negative controls were established by replacing the primary antibody with goat serum. On completion of incubation the sections were washed and probed with anti-rabbit secondary antibody labelled with horseradish peroxidase for 30 min (ABC-Elite Rabbit IgG, Vectastain Elite Kit, PK-6101, Vectastain ABC Kit, Vector, Peterborough, UK). FOXO3 was detected using 3, 3′-diaminobenzidine (DAB) peroxidase substrate kit (Vector Laboratories Ltd, Peterborough, UK). Follicles were scored positive (activated) when brown staining was observed in the ooplasm and negative (non-growing) when brown staining was observed in the germinal vesicle.

### Western blotting

Protein was lysed in RIPA buffer with Protease and Phosphatase Inhibitors (Roche Products Limited, Welwyn Garden City, UK). Samples (20 μg) were denatured and loaded onto 4–20% gels in Tris-Hepes running buffer with 5 μl PageRuler Plus ladder (Thermo Fisher Scientific, Hemel Hempstead, UK) and run at 100 volts for ∼1 h. Immobilon-FL blotting paper (Merck Millipore, Nottingham, UK) was soaked in methanol, rinsed in distilled water (dH2O) and soaked in Semi-Dry Transfer Buffer (Thermo Fisher Scientific). The gel was also equilibrated in dH20 and then Semi-Dry Transfer Buffer before transfer for 9 min on a Pierce Fast Semi-Dry Blotter (Thermo Fisher Scientific).

Blots were rinsed in dH20 and blocked in Blocking Buffer (Rockland Immunochemicals, Inc. PA, USA) 1:1 in phosphate buffered saline plus 0.1% Tween (PBS-T) for 1 h at room temperature. Blots were then incubated with a rabbit polyclonal antibody raised against either Akt (9272) or pAkt (9271) (Cell Signaling, both diluted 1:1000) and with a mouse monoclonal antibody raised against beta-actin (Sigma-Aldrich; antibody A5441, diluted 1:5000) overnight at 4°C. Blots were washed in 0.1% PBS-T and then incubated with a polyclonal donkey antibody raised against rabbit IgG (heavy and light chain) conjugated with Alexa Fluor680 (Invitrogen, Paisley, UK; diluted 1:10000) and a donkey polyclonal antibody raised against mouse IgG (H&L) conjugated with IRDye800 (Rockland Immunochemicals; diluted 1:10000) for 1 h at room temperature. They were then washed in PBS-Tween and then PBS before scanning using a LI-COR Odyssey (LI-COR Biosciences UK Ltd, Cowley Road, Cambridge, UK).

### Evaluation of histology

A light microscope with a crossed micrometer was used to examine every section of each fragment of cortical tissue. Using a slightly modified version of the system employed by Telfer *et al.* (2008) follicles were classified according to their developmental stage dependent on the morphology and abundance of the granulosa cells observed as follows: (i) non-growing follicles: follicles constituting the resting pool, characterized by oocytes surrounded by a complete or incomplete single layer of cells, either all flat (primordial) or a mixed layer of flattened and cuboidal cells (transitory) (Smitz and Cortvrindt, 2002), (ii) primary follicles: follicles comprising of an oocyte surrounded by a single layer of cuboidal granulosa cells, (iii) secondary follicles: multilaminar follicles with more than a single layer of cuboidal granulosa cells and (iv) antral: antrum present within a multilaminar follicle. Evaluation of isolated follicles and follicles within cortical fragments was made on the section containing the nucleolus except when determining the presence of an antrum in isolated follicles where all sections of the follicle were examined. Mean follicle and oocyte diameter was recorded for each isolated and fragment-enclosed follicle evaluated.

Follicle morphological health was determined by assessment of oocyte appearance and granulosa cell pyknosis on histological sections of tissue fragments. Follicles were assessed using the cross-section containing the nucleolus. Oocyte and granulosa cell morphology were assessed as described by McLaughlin and Telfer (2010) and McLaughlin *et al.* (2010) with slight modification. Briefly for follicles to be categorized as morphologically normal, the oocyte must be grossly circular, surrounded by a zona pellucida, have a visible germinal vesicle and defined nucleolus with <10% of pyknotic granulosa cells present; follicles meeting these criteria were considered to have survived the *in vitro* period.

### Statistical analyses

Inter and intra-treatment differences in follicle and oocyte diameters were compared by one-way ANOVA with subsequent *t*-tests. Proportional data were compared using chi-squared analysis and Fishers Exact test.

## Results

### Assessment of cortical tissue pieces

**Figure 1:**
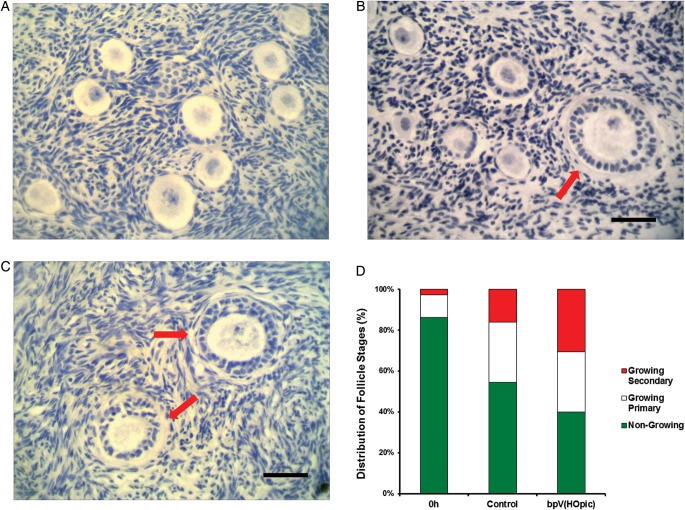
(**A**) Photomicrograph of 0 h human ovarian cortex; all follicles are at the earliest stages of development. (**B** and **C**) Photomicrographs of cultured ovarian cortex at Day 6 showing activation of follicle growth coincident with non-growing follicles in control (B), and in bpV(HOpic) treated cortical tissue (C) showing multiple secondary follicles. (**D**) Distribution of follicles in adult human ovarian cortical tissue by stage of development in uncultured tissue (0 h) and at Day 6. Follicle distribution is shown as a percentage of the total. Arrows indicate *in vitro* grown secondary follicles. Scale bar = 50 microns.

The number and developmental stage of follicles in human ovarian cortex at 0 h was determined in 54 freshly fixed cortical tissue pieces of which 28 contained follicles; a total of 152 follicles were identified and analysed. The number of follicles observed varied considerably within and between patients in both obstetric and gynaecological tissue pieces ranging from 0 to 22 follicles. This highly irregular distribution of follicles in human ovarian cortex has been reported previously (Block 1951; Hreinsson *et al.*, 2002; Poirot *et al.*, 2002; Schmidt *et al.*, 2003; Kristensen *et al.*, 2011). Of the 152 follicles analysed, 86.2% were non-growing with the remaining follicles being at the primary (11.1%) or secondary stage (2.6%). No antral follicles were observed in any uncultured tissue fragments (Fig. 1A). No difference was observed in the maturity of follicles between obstetric and gynaecological tissue pieces.

### Follicle activation in cultured cortical fragments

Microscopic examination of cultured cortical fragments showed that after 6 days incubation significant initiation of follicle growth had occurred in both treatment groups compared with uncultured tissue. 45.5 and 60% of follicles were observed to be growing in control and bpV(HOpic) exposed tissue, respectively (86 of 189 follicles in control compared with 187 of 312 follicles in bpV(HOpic)). Significantly more growing follicles were observed in bpV(HOpic) treatment compared with control (*P* = 0.0016). The most mature follicles present in either of the treatments were secondary follicles (Fig. 1B and C), with a significantly greater percentage present in tissue exposed to bpV(HOpic) compared with control (30.5 versus 16%) (*P* = 0.012; Fig. 1D). No antral follicles were observed in either treatment group after 6 days *in vitro*.

### Activation of PI3K pathway in bpV(HOpic) exposed tissue

**Figure 2:**
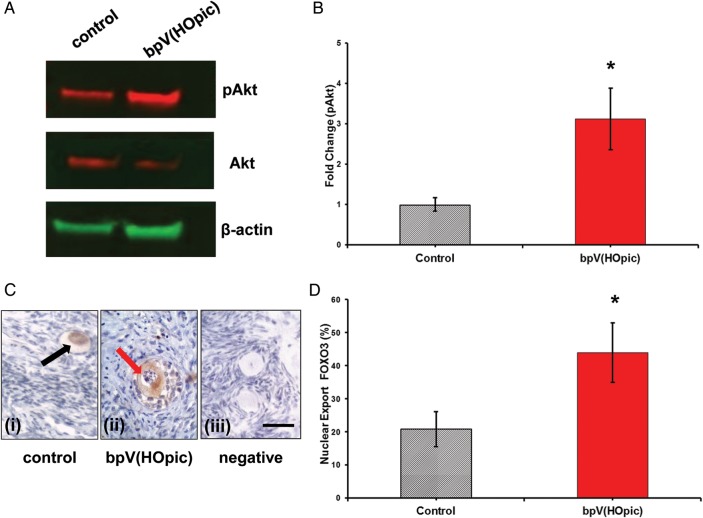
(**A**) Western blot showing enhanced phosphorylation of Akt in tissue exposed to bpV(HOpic) compared with control cultured tissue, indicating PTEN inhibition is associated with increased activation of the PI3K pathway; β-actin used as loading control. (**B**) The fold change in expression of pAkt in human ovarian tissue in control and bpV(HOpic) treated tissue, indicating significantly higher AKT phosphorylation in bpV(HOpic) treated tissue (*P < 0.01, *n* = 5). (**C**) Photomicrographs showing immunohistochemical detection of FOXO3 in human ovarian cortex. (i) Brown staining indicating nuclear enclosed, inactivated FOXO3 in a non-growing follicle exposed to control medium. Arrow indicates discrete brown staining in germinal vesicle; (ii) export of FOXO3 from the nucleus indicated by brown staining in the ooplasm of *in vitro* grown secondary follicle exposed to bpV(HOpic), indicating activation of PI3K pathway with Akt phosphorylation. Arrow indicates absence of staining in the germinal vesicle. (iii) Negative control. Scale bar = 30 microns. (**D**) Oocyte nuclear export of FOXO3 in control and bpV(HOpic) treated tissue: a significantly greater percentage of Oocytes showed FOXO3 nuclear export in tissue exposed to bpV(HOpic) compared with control (*P = 0.0019). Eighty-nine follicles analysed in total, 48 from control and 41 from bpV(HOpic) exposed tissue.

To investigate whether the PI3K pathway was influenced by suppression of PTEN in human ovarian cortex, western blotting was performed on control and bpV(HOpic) exposed tissue fragments (*n* = 46 and 51, respectively) from 8 of the 17 patients included in this study; the number varied between patients owning to differing size of biopsies. A minimum of four and a maximum of eight fragments per treatment were used per western blot. Figure 2A shows western blotting indicating an increase in pAkt in bpV(HOpic) exposed tissue compared with control. Quantification indicated that Akt phosphorylation was increased ∼3-fold in tissue exposed to bpV(HOpic) for 24 h compared with control (*P* = 0.0086, *n* = 5 independent blots) (Fig. 2B). Thus PTEN suppression induced by bpV(HOpic) results in increased PI3K pathway activation.

### Nuclear exclusion of FOXO3

Immunohistochemistry was performed to determine whether suppressing PTEN resulted in nuclear exclusion of FOXO3, a downstream protein component of the PI3K pathway which exits the nucleus as non-growing follicles are activated (Li *et al.*, 2010). Tissue sections incubated with and without bpVHOpic from 10 patients were examined; treatment groups were blinded to the examiner. A total of 89 follicles were analysed (control *n* = 48; bpV(HOpic) *n* = 41). The number of sections per slide and of follicles per section was highly variable, characteristic of follicle distribution in human ovarian tissue. Non-nuclear detection of FOXO3 was observed in significantly more follicles (43.9%) in tissue exposed to bpV(HOpic) compared with controls (20.8%, *P* = 0.019; Fig. 2C and D). In control exposed tissue FOXO3 exclusion was observed in both primary and secondary growing follicles (80% primary and 20% secondary). Similarly in bpV(HOpic) exposed tissue FOXO3 exclusion was observed in both primary and secondary follicles (45% primary and 55% secondary).

### Follicle survival in cultured cortical fragments

**Figure 3:**
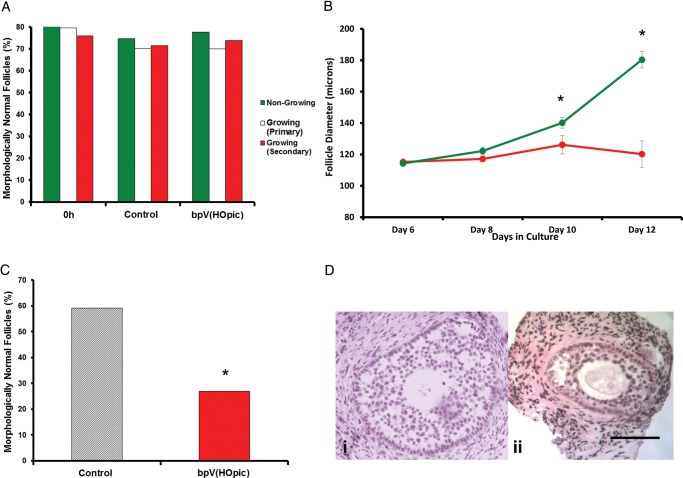
(**A**) The percentage of morphologically normal follicles observed in tissue fragments in uncultured tissue (0 h) and after 6 days culture in control or bpV(HOpic) medium. Percentages of healthy follicles are shown by developmental stage. (**B**) Mean diameter (µm) of follicles isolated from control (green) and bpV(HOpic) (red) exposed tissue over a further 6 days *in vitro*. At the end of the culture period follicles isolated from control tissue were significantly larger than bpV(HOpic) treated follicles (*P < 0.001). (**C**) On completion of the isolated follicle culture period a significantly greater percentage of control exposed follicles were morphologically normal compared with those exposed to bpV(HOpic) (*P < 0.01). (**D**) Photomicrographs of isolated follicles after a total of 12 days in culture. (i) Morphologically normal IVG follicle from control cultured tissue, and (ii) morphologically abnormal IVG follicle isolated from bpV(HOpic) exposed tissue. Scale bar = 50 microns.

Follicle morphological health was determined by assessment of oocyte appearance and granulosa cell pyknosis on histological sections of tissue fragments. A non-significant reduction in survival was observed between both treatment groups for all stages of follicle development compared with uncultured controls (Fig. 3A). Non-growing follicles had a high *in vitro* survival rate irrespective of treatment with 74.7 and 77.6% of follicles appearing morphologically normal following incubation in control and bpV(HOpic), respectively, compared with 80.1% in uncultured tissue (non-growing versus control *P* = 0.601; non-growing versus bpV(HOpic) *P* = 0.655). No significant difference was observed in primary or secondary follicle survival between control and 1 µM bpV(HOpic) with over 69% of follicles from both treatments appearing morphologically normal after 6 days incubation (primary follicles, control versus bpV(HOpic) *P* = 0.934; secondary follicles, control versus bpV(HOpic) *P* = 0.759) (Fig. 3A).

### Growth of isolated follicles

Care was taken during mechanical isolation of follicles to ensure basal laminae were not exposed to prevent compromising follicle development. Due to the considerable variation in stromal tissue density between and within biopsies the amount of tissue surrounding isolated follicles varied; however, sufficient stromal tissue was left enclosing each follicle to provide a presumptive theca cell layer for further follicular development. On completion of the first step of the culture system there was no significant difference in the diameter of follicles isolated from bpV(HOpic) exposed tissue compared with those isolated from control (Fig. 3B). Follicle diameters were recorded every second day after isolation until completion of the culture period. Measurements were recorded using a dissecting microscope with a crossed micrometer. During the subsequent 6 day culture period follicles isolated from control tissue increased significantly in diameter (114 ± 2 to 181 ± 8 µm; *P* < 0.001), whereas follicles isolated from tissue exposed to bpV(HOpic) exhibited limited, nonsignificant growth (115 ± 1 to 120 ± 3 µm; Fig. 3B). Thus at the end of this period follicles from control tissue were significantly larger than those from bpV(HOpic) treated tissue (*P* = 0.0001).

### Survival of isolated follicles

Survival of isolated follicles was assessed as per the criteria used for evaluation of follicle survival within cultured tissue fragments described above. Three follicles degenerated during the first 4 days of individual culture; 1 from tissue exposed to control medium and 2 from tissue fragments treated with bpV(HOpic); data from these follicles were excluded from *in-vitro* growth analysis. Follicular morphology was assessed by inspection of histological sections and was used to determine normality and thus survival. Follicle survival was significantly higher in follicles from tissue fragments exposed to control medium, with 13 of the 22 (59%) follicles surviving the culture period being deemed morphologically normal compared with 7 of 26 (27%) surviving follicles from tissue fragments exposed to bpV(HOpic) (*P* = 0.02; Fig. 3C). Morphological abnormalities observed included shrunken oocytes, widespread granulosa cell pyknosis and loss of granulosa cell-oocyte proximity. Representative images of both normal and morphologically abnormal follicles are shown in Fig. 3D.

## Discussion

Disruption of the PI3K pathway using knockout models (Fan *et al.*, 2008; Reddy *et al.*, 2008) and pharmacological stimulators and/or inhibitors (Li *et al.*, 2010; Adhikari *et al.*, 2012) promotes activation of follicle growth in mice and xeno-transplanted human ovarian tissue and enhances murine ovulation. In this study we investigated the ability of bpV(HOpic) a pharmacological inhibitor of PTEN, a major negative regulator of the PI3K pathway, to affect human ovarian follicle activation and development in both tissue fragments and isolated follicles *in vitro*. We hypothesized that using an established two-step culture system (Telfer *et al.*, 2008) selective promotion of the PI3K pathway by pharmacological inhibition of PTEN would promote (i) initiation of growth in quiescent follicles within cortical tissue fragments and (ii) the development of *in vitro* grown (IVG) secondary follicles to the large pre-antral and/or early antral stage. Our results show that exposure of human ovarian cortex to bpV(HOpic) increased the activity of the PI3K pathway and promoted follicle activation and development to the secondary stage. However, following isolation from cortical tissue fragments the key novel finding of this study emerged, that secondary follicles previously exposed to bpV(HOpic) grew very poorly and their survival was severely compromised.

In this study increased activity of the PI3K pathway was confirmed by an increase in Akt phosphorylation and nuclear export of FOXO3. Whilst within the germinal vesicle, FOXO3 is a recognized suppressor of primordial follicle growth; when relocated to the ooplasm this suppression is lifted and follicle activation occurs (Gonzalez-Robayna *et al.*, 2000). Li *et al.* (2010) also demonstrated nuclear export of FOXO3 in the oocytes of human ovarian follicles; however, a recent study has challenged this paradigm and suggests that FOXO3 is not a requirement for human primordial follicle growth arrest (Tarnawa *et al.*, 2013). Tarnawa and colleagues reported on data obtained from one human ovary whereas in this study we analysed immunohistochemical detection of FOXO3 exclusion from 10 patients. Whilst is unclear how many ovaries Li *et al.* (2010) included in their study they also describe results from multiple human biopsies; therefore we suggest the divergence of results may be due to the difference in the number biopsies analysed.

The controlled promotion of human ovarian follicle development to obtain oocytes capable of undergoing *in vitro* maturation and fertilization with subsequent embryo development is the ultimate goal of human ovarian follicle culture. Pharmacological promotion of follicle growth *in vitro* followed by a xeno-transplantation model has resulted in embryo development and live litters being achieved in mice (Li *et al.*, 2010; Adhikari *et al.*, 2012). The initial results presented here are in agreement with previous work demonstrating that pharmacological inhibition of PTEN stimulates follicle activation in the human ovarian cortex (Li *et al.*, 2010). In our study a concentration of 1 µM was chosen because in preliminary experiments using 10 and 100 µM bpV(HOpic), increased follicle growth was associated with gross morphological abnormalities in activated follicles prior to isolation from cortical fragments (not shown). We suggest that the reduced follicle survival observed with higher concentrations of bpV(HOpic) treatment is associated with the specific manner in which tissue is prepared for incubation. Unlike tissue cubes, our method of tissue fragment preparation maximizes the exposure of the ovarian cortex to the culture medium by loosening the cortical surface and removing excess dense connective stromal tissue which may prevent cortical exposure to media components and physically impede follicle activation and development. The present method of tissue preparation has been successfully employed to promote the activation of follicle growth in bovine and in fresh and cryopreserved-thawed pre-pubertal as well as pubertal and adult human ovarian tissue (Telfer *et al.*, 2008; McLaughlin and Telfer, 2010; Anderson *et al.*, 2014). We suggest that preparing the cortex in this manner allows maximum cortical exposure to the culture medium, and in preliminary experiments determined that the optimal concentration of bpV(HOpic) which promoted activation of follicle growth and maintained normal morphology was 1 µM. It is unclear whether the morphological normality of human follicles activated by higher doses of bpV(HOpic) was assessed prior to xeno-transplantation (Li *et al.*, 2010). It is however noteworthy that Adhikari *et al.* (2012) exposed mouse ovaries to 1 µM bpV(HOpic) to produce fertilizable IVG oocytes (Adhikari *et al.*, 2012). bpV(HOpic) activated follicles were intact after 6 days incubation and not different in diameter from controls. bpV(HOpic) treatment did not therefore recruit a suboptimal follicle population. Following a further 6 days in culture bpV(HOpic)-exposed follicles grew poorly and deteriorated significantly. A previous study reported normal follicular development in mice following a conditional deletion of the *PTEN* gene in oocytes with normal oocyte maturation, fertility and litter size observed (Jagarlamudi *et al.*, 2009). However our study is not the first report of bpV(HOpic) treatment affecting human ovarian follicle development deleteriously. Lerer-Serfaty *et al.* (2013) recently reported widespread follicle destruction in cryopreserved-thawed human tissue exposed to a high concentration of 100 µM bpV(HOpic); this supports the view that manipulation of the PI3K pathway may also have negative impact on other signalling mechanisms (Blanco-Aparicio *et al.*, 2007). Whilst PI3K activation and subsequent pAkt promotion by bpV(HOpic) can induce follicle activation in human ovarian tissue, it is possible that other Akt-independent effects of PTEN inhibition cause the follicle degeneration seen in this study and by other investigators *(*Lerer-Serfaty *et al.*, 2013).

Growth was limited and normal follicular morphology significantly compromised in follicles isolated from tissue fragments exposed to bpV(HOpic). It is well established that maintenance of bi-directional oocyte/somatic cell communication is vital for normal follicle development (Albertini *et al.*, 2001; Eppig, 2001) and although an extensive morphological analysis was not possible due to degeneration, it is suggested that vital oocyte-somatic cell contact was damaged as a result of bpV(HOpic) exposure. As activated follicles exposed to bpV(HOpic) were morphologically normal within tissue fragments at Day 6 of the culture period, it is possible that the surrounding stromal environment was able to modulate or circumvent the deleterious effects of PTEN inhibition over a relatively short period of time. It is unlikely however that allowing bpV(HOpic) activated follicles to remain within the presumptive protection of the stromal fragments would result in the development of morphologically mature follicles as it has been demonstrated that prolonged culture results in widespread follicle loss by atresia (Hovatta *et al.*, 1999).

A human live birth has recently been reported following treatment of ovarian tissue for 48 h *in vitro* with bpV(HOpic) and 740Y-P, an Akt stimulant, followed by replacement and IVF (Kawamura *et al.*, 2013). This is an encouraging development but is difficult to compare directly to the results reported here which utilize an entirely *in vitro* system. The human tissue Kawamura *et al.* (2013) incubate with Akt stimulants is described as containing a range of both non-growing and growing follicles; the early oocytes retrieved from pre-ovulatory follicles were believed to be derived from rapidly growing secondary follicles contained in the auto-grafted tissue. Exposure to bpV(HOpic) and 740Y-P may confer direct or indirect protection from apoptosis on secondary follicles, although the absence of control experiments does not allow clear assessment of the effects of the Akt stimulants in that study. The present study does not investigate this possibility as only follicles <40 µm in diameter, i.e. non-growing or primary are present at the time of tissue exposure to bpV(HOpic).

In conclusion these data confirm that the PI3K pathway is involved in the regulation of initiation of growth of human ovarian follicles. Treatment of human ovarian cortical tissue with bpV(HOpic) promotes activation and development of follicles but does not confer any advantage on follicle development beyond the secondary stage and does not prolong isolated follicle survival, indeed a significant deleterious effect was found.

It does not appear therefore that promotion of the PI3K pathway by inhibition of PTEN using bpV(HOpic) is a candidate treatment for the robust generation of numbers of good-quality mature IVG human oocytes. Pharmacological manipulation of the PI3K pathway to promote follicle development has been successfully achieved in mice via promotion of other components of PI3K, e.g. by using 740Y-P (Li *et al.*, 2010), and it remains to be seen whether this molecule can be used to promote follicle activation and maintain morphologically normal growth in human ovarian follicles.

## Authors' roles

M.M.: conception and design of study, experimental work and acquisition of data, analysis and interpretation of data, manuscript preparation, final approval of manuscript. H.L.K.: experimental work and acquisition of data, analysis and interpretation of data, manuscript preparation, final approval of manuscript. R.A.A.: conception and design of study, analysis and interpretation of data, funding for study, manuscript preparation, final approval of manuscript. E.E.T.: conception and design of study, analysis and interpretation of data, funding for study, manuscript preparation, final approval of manuscript.

## Funding

Funded by MRC grants G0901839 and G1100357. Funding to pay the OpenAccess publication charges for this article was provided by the University of Edinburgh.

## Conflict of interest

None declared.
